# Light source optimization for partially coherent holographic displays with consideration of speckle contrast, resolution, and depth of field

**DOI:** 10.1038/s41598-020-75947-0

**Published:** 2020-11-02

**Authors:** Seungjae Lee, Dongyeon Kim, Seung-Woo Nam, Byounghyo Lee, Jaebum Cho, Byoungho Lee

**Affiliations:** School of Electrical and Computer Engineering, Seoul National University, Gwanak-gu Gwanakro 1, Seoul, 08826 Republic of Korea

**Keywords:** Displays, Lasers, LEDs and light sources

## Abstract

Speckle reduction is an important topic in holographic displays as speckles not only reduce signal-to-noise ratio but also possess an eye-safety issue. Despite thorough exploration of speckle reduction methods using partially coherent light sources, the trade-off involved by the partial coherence has not been thoroughly discussed. Here, we introduce theoretical models that quantify the effects of partial coherence on the resolution and the speckle contrast. The theoretical models allow us to find an optimal light source that maximizes the speckle reduction while minimizing the decline of the other terms. We implement benchtop prototypes of partially coherent holographic displays using the optimal light source, and verify the theoretical models via simulation and experiment. We also present a criterion to evaluate the depth of field in partially coherent holographic displays. We conclude with a discussion about approximations and limitations inherent in the theoretical models.

## Introduction

Near-eye display technologies have been widely advertised and recognized as a next-generation display platform that delivers an unprecedented immersive experience. With increasing commercial and economic demands in augmented and virtual reality^1^, it has become an important topic to develop a daily usable near-eye display system. However, it is challenging to realize a near-eye display system that satisfies all commercial demands. Customers expect a more immersive display device with a larger field of view, higher resolution, and faster frame rate. Besides, they prefer a more comfortable display device that is lightweight, wearable, and free from vergence-accommodation conflict. Recently, various researches related to holographic near-eye displays^2–4^ have been introduced to show a new possibility of realizing an ultimate near-eye display. The intrinsic optical principle of holographic displays, wavefront modulation using a coherent light source, carries several advantages for near-eye displays compared to other approaches including multi-plane displays^5–8^, light field displays^9,10^, volumetric displays^11–13^, and gaze-contingent displays^14^.

Holographic displays modulate a coherent illumination’s complex amplitude to reconstruct arbitrary wavefront using a spatial light modulator (SLM). The combination of the coherent illumination and the SLM enables the displays to reconstruct volumetric objects, and corrects the optical aberration accompanied by optical elements^3^. Holographic displays are versatile, which have been studied from several perspectives: display applications including near-eye displays^2–4^, tabletop displays^15,16^, and projection-type displays^17^; spatial-bandwidth product improvement using a scattering^18^ or non-periodic^19^ medium; and speckle analysis of holographic displays according to light sources^20^. In the near-eye display field, a lightweight glasses-like optical design was introduced^3,4,21^, which supports a large field of view, high resolution, and accurate focus cues. Despite the remarkable advantages of holographic near-eye displays, it is not negligible that there is a drawback of utilizing the coherent light source for the wavefront modulation. The major drawback is coherent interference (i.e., speckle), which degrades the signal-to-noise ratio and could be hazardous for the human visual system^20,22^.

Speckle can be interpreted as a randomly generated pattern via constructive or destructive interference. As the speckle intensity is randomly determined, speckle might distort the original signal and reach a hazardous intensity level for the human visual system. Unfortunately, speckle is inevitably generated at the observer’s retina in holographic near-eye displays. Although various approaches^20,23–25^ have been introduced for speckle reduction, most speckle reduction methods accompany the sacrifice of spatial resolution or frame rate. Speckle reduction methods generally exploit the optical or temporal superposition of independent speckle patterns. The optical superposition corresponds to applying a partially coherent light source for speckle reduction. The partially coherent light source includes a bundle of mutually incoherent light sources^26,27^, an optical fiber^28,29^, and spectrally-broadband light source (e.g. a light-emitting diode, LED)^25,30–34^. The temporal superposition is to update speckle patterns at a fast frame rate. Speckle patterns are updated by refreshing computer-generated holograms (CGHs)^23,35,36^ or exploiting mechanical movement of optical elements (e.g. moving diffuser)^24,37–40^. Despite numerous researches on speckle reduction of coherent illumination, there has not been a thorough and quantitative discussion about the trade-off between speckle and spatial resolution in holographic near-eye displays.

Here, we introduce theoretical models that depict how the light source’s characteristics affect speckle contrast and spatial resolution. Using the theoretical models, we optimize the light source characteristics using a gradient decent method. The optimization finds a light source that reduces the speckle contrast while minimizing the sacrifice of resolution and depth of field. We also demonstrate a prototype of partially coherent holographic near-eye displays using an optimal light source. Secondly, we verify the theoretical model used for light source optimization by simulation and experiment. For the experimental validation, we compare various light sources with different angle and wavelength diversity. Lastly, we introduce a criterion to evaluate the depth of field where high-resolution images can be reconstructed with a certain level of speckle reduction. We note the inevitable sacrifice of the depth of field or resolution for considerable speckle reduction even with the light source optimization. To alleviate the drawback of using a partially coherent light source, we conceive a methodology that applies a gaze-contingent approach^14^ to holographic displays. We conclude with a discussion about approximations and limitations in the theoretical models.

## Results

### Background of partially coherent holographic displays

**Figure 1:**
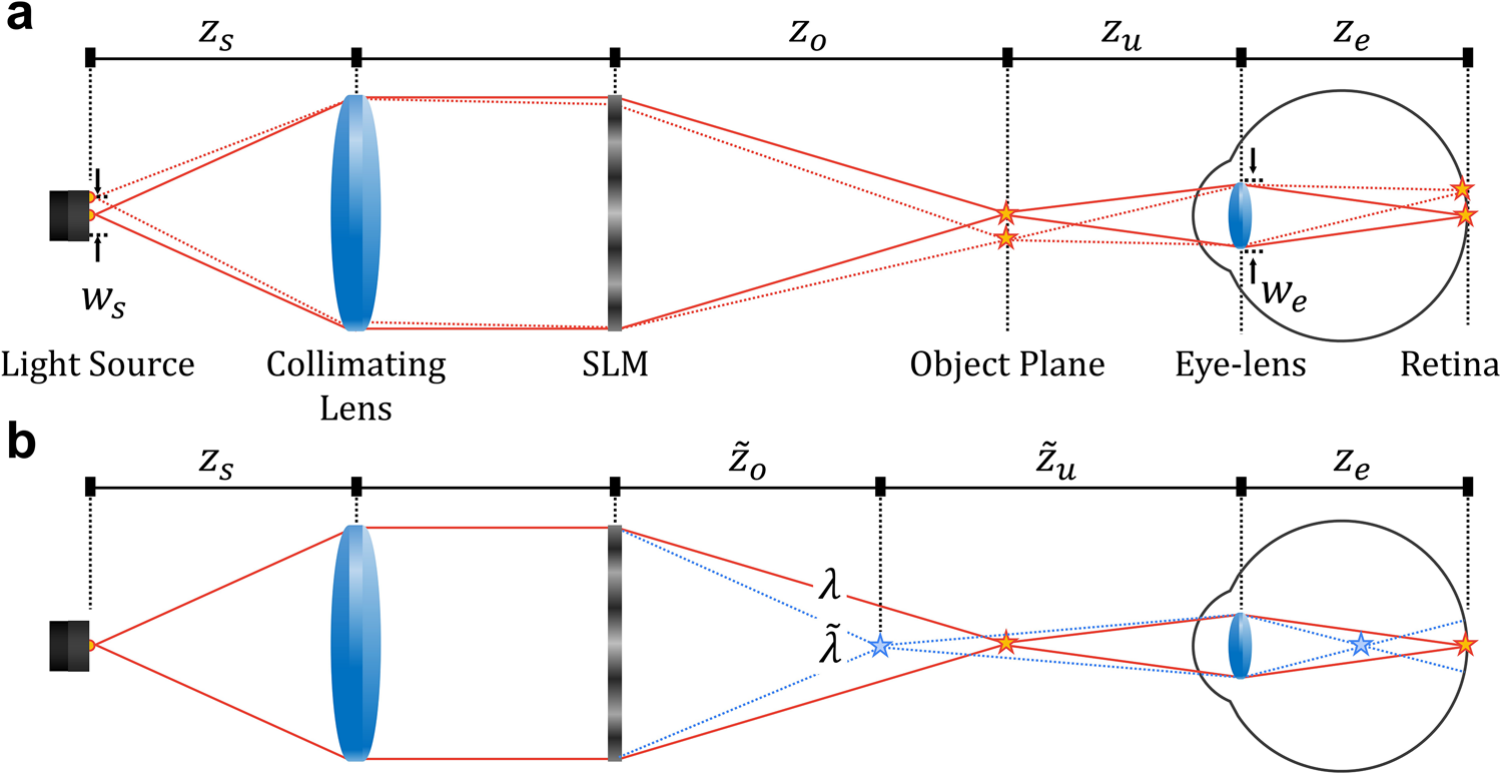
Illustration of the lateral and axial shifts of holographic images according to (**a**) angle diversity and (**b**) wavelength diversity. We suppose a complex amplitude transmission coefficient is given by an SLM to reconstruct a star at a depth of $z_o$. (**a**) Dot lines describe how the angle diversity laterally shifts the reconstructed star. (**b**) Blue lines describe how the reconstructed star is axially shifted by the wavelength diversity ($\lambda > {\tilde{\lambda }}$).

It has been well studied that increasing angle or wavelength diversity of light sources contributes to independent speckle generation and speckle reduction^31,33^. Although these methods are applicable for holographic displays, we need to recall that angle or wavelength diversity involves the decline of spatial resolution^22^. The reduction of speckle contrast usually sacrifices the resolution in partially coherent holographic displays. To comprehend this interrelation, we investigate the optical effect of angle or wavelength diversity in holographic displays. As illustrated in Fig. 1, angle or wavelength diversity leads the holographic images to be reconstructed with undesired shift. The angle diversity contributes to the lateral shift of holographic images, while the wavelength diversity contributes to the axial shift. The lateral and axial shifts may generate independent speckles at the retina, which results in the speckle reduction. However, these shifts also make the observed images more blurry, and degrade the spatial resolution of holographic displays. This qualitative model gives an intuition to understand a trade-off in partially coherent holographic displays.

Our goal is to formulate theoretical models that enable a quantitative analysis of the trade-off between the resolution and the speckle contrast. More specifically, our interest is in the analysis of a holographic near-eye display system illustrated in Fig. 1. In this system, a user observes holographic images reconstructed by an ideal SLM that modulates the complex amplitude of a collimated light wave. Note that the ideal SLM is substituted with a phase-only or amplitude-only SLM using complex encoding^41^. The light source has a square aperture whose width and apodization function are $w_s$ and *p*(*x*). The light source is collimated by a lens with a focal length of $z_s$. The ratio of $w_s$ to $z_s$ determines the angle diversity *a*(*x*) while the spectral power distribution (SPD) $M(\lambda )$ of the light source determines the wavelength diversity. The SPD of the light source has the central wavelength of $\lambda _o$. The ideal SLM at $z=0$ is supposed to reconstruct a diffusive object at depth of $z=z_o$, where the diffusive surface is represented by a random phase modulation. We assume the user’s observation system is a simple optical system divided into the eye-lens with a circular pupil of diameter $w_e$ and the retina. The distances among the object, the eye-lens, and the retina are denoted by $z_u$ and $z_e$ as shown in Fig. 1.

### Resolution analysis model

For the resolution analysis, the theoretical model assumes that the spatial coherence of the light source is limited. With this assumption, the illumination with angle and wavelength diversity is interpreted as an incoherent superposition of wavelets with different wave vectors and wavelengths. If partially coherent holographic displays reconstruct a point, a user observes superposition of holographic images reconstructed by the wavelets. Each wavelet with a specific wave vector and wavelength reconstructs a shifted point and all reconstructed holographic images are incoherently added up in the observation plane. To analyze how shifted objects are synthesized at the observation plane, we first add up the intensity of laterally shifted objects reconstructed by wavelets with a wavelength of ${\lambda }_s$. The intensity sum of the objects at the depth of $z = \lambda _o {z_o}/ {\lambda }_s$ is the convolution of the original object and the angle diversity function $a({\mathbf{x}})$. Thus, the Fourier transform of the intensity sum is the multiplication of Fourier transforms of angle diversity function $A({\mathbf{v}})$ and the original object image $I_o({\mathbf{v}})$. Note that the angle diversity function $a({\mathbf{x}})$ is given by $p_s(z_o{\mathbf{{x}}}/z_s)$, where $p_s({\mathbf{x}})$ is the light source aperture’s apodization function.

Second, the intensity sum at the depth of $z = \lambda _o {z_o}/ {\lambda }_s$ is transferred to the observation plane. Using the optical transfer function $T({\mathbf{{v}}};{{\lambda }_s})$^42^, we calculate the Fourier transform of the observation image $R_A$ reconstructed by single wavelength, which is given by

1 $$\begin{aligned} {R_A} {\left( \frac{{z_u}}{z_e}{} {\mathbf{{v}}};{{ \lambda }_s} \right) } = T({\mathbf{{v}}};{{ \lambda }_s})A({\mathbf{{v}}};{{ \lambda }_s}){I_o}({\mathbf{{v}}}), \end{aligned}$$

where $z_u/z_e$ is the magnification ratio of the eye-lens in the imaging geometry. Third, we integrate the observation images according to the wavelength diversity of the light source given by the SPD $M(\lambda _s)$. The Fourier transform of the resultant intensity *R* is given by

2 $$\begin{aligned} \begin{aligned} R {\left( \frac{z_u^c}{z_e}{} {\mathbf{{v}}} \right) } \approx {I_o}({\mathbf{{v}}})\int _{{\lambda _s}} M({\lambda _s}) T({\mathbf{{v}}};{\lambda _s}) {A({\mathbf{{v}}};{\lambda _s}) d{\lambda _s}} = {T_h}({\mathbf{{v}}}){I_o}({\mathbf{{v}}}), \end{aligned} \end{aligned}$$

where $z_u^c$ is the average distance between the eye-lens and the reconstructed object. Note that we suppose on-axis imaging geometry where the magnification variation effect according to the wavelength change is negligible. Finally, the transfer function of partially coherent holographic displays is given by abs$(T_h)$. The transfer function model allows us to analyze the resolution of holographic displays with consideration for both of the angle and wavelength diversity. Detailed derivation of the equations in this section is described in Supplementary Material.

### Speckle analysis model

Speckles are observed in an additive composition of numerous signals, each of which has independent complex amplitude^22^. When each complex amplitude has random amplitude and phase, the additive composition of those signals is interpreted as a “random walk.” In other words, the resultant intensity might be large or small according to the sum of the various signals with different relative phases. The first-order statistical properties of the speckle intensity is described based on probability theory. If the random phase of each complex amplitude is uniformly distributed on $(-\pi , \pi )$ where fully developed speckle appears^22^, the complex amplitude of the signal sum follows Rayleigh probability density. Using this probability density function, we can predict the intensity distribution of the signal sum as follows.

3 $$\begin{aligned} {p_I}(I) = \frac{1}{{{m_I}}}\exp \left( { - \frac{I}{{{m_I}}}} \right) , \end{aligned}$$

where $p_I(I)$ is the probability density function for the intensity, and $m_I$ is the mean intensity. To estimate the strength of speckle intensity fluctuation, we usually measure speckle contrast (*C*) and signal-to-noise ratio (SNR) which are given by

4 $$\begin{aligned} C = \frac{{{\sigma _I}}}{{{m_I}}}, \quad SNR = \frac{{{m_I}}}{{{\sigma _I}}}, \end{aligned}$$

where $m_I$ and $\sigma _I$ are mean and standard deviation of the intensity, respectively. Note that the standard deviation ($\sigma _I$) is identical with the mean intensity ($m_I$) when fully developed speckles appear. Thus, these equations show that the SNR of coherent imaging with fully developed speckles is 1, which indicates a severe noise.

In partially coherent holographic displays, the observed speckle pattern is a sum of speckle patterns generated by mutually incoherent wavelets. The sum of speckle patterns is described by

5 $$\begin{aligned} \begin{aligned} {I_s} = \sum \limits _{j = 1}^{{N_\lambda }} {\sum \limits _{i = 1}^{{N_a}} {M({\lambda _i})a({{\mathbf{{x}}}_j}){I_{ij}}\Delta \lambda \Delta {\mathbf{{x}}}} }, \end{aligned} \end{aligned}$$

where $N_\lambda$ and $N_a$ are the sampling numbers of wavelength and angle diversity, respectively. $I_s$ is the total intensity of speckles. $I_{ij}$ is a speckle generated by a wavelet of the wavelength $\lambda _i$, which comes from ${\mathbf{x}}$ on the aperture. Note that $I_{ij}$ is random variable that reflect characteristics of fully developed speckles where $m_I=\sigma _I$ and $C=1$. For more simplified notations, we represent the two variables for angle and wavelength diversity by single vector ${\mathbf{t}}$. The simplified equation is given by

6 $$\begin{aligned} \begin{aligned} {I_s} = \sum \limits _{k = 1}^{{N_\lambda }{N_a}} {c({{\mathbf{{t}}}_k}){I_k}\Delta {{\mathbf{{t}}}_k}}, \end{aligned} \end{aligned}$$

where $c({{\mathbf{{t}}}})$ is a function designed to represent $M({\lambda })a({{\mathbf{{x}}}})$. From the Eq. (6), we derive the speckle contrast of the superposed speckles $C_s$ given by

7 $$\begin{aligned} \begin{aligned} C_s = \frac{{\sqrt{\sum \nolimits _j {\sum \nolimits _i {{\mathop {\hbox {corr}}\nolimits } ({I_i},{I_j})c({\mathbf{{t}}_i})c({\mathbf{{t}}_j})\Delta {\mathbf{{t}}_i}\Delta {\mathbf{{t}}_j}} } } }}{{\sum \nolimits _i {c({\mathbf{{t}}_i})\Delta {\mathbf{{t}}_i}} }}. \end{aligned} \end{aligned}$$

To calculate the correlation between two speckles, ${{\mathop {\hbox {corr}}\nolimits } ({I_i},{I_j})}$, we first consider the influence of angle diversity on the speckle decorrelation. Similar to the lateral shift of the reconstructed object, the angle diversity leads to the speckle displacement at the observation plane^43^. If the speckle displacement is larger than the speckle size at the observation plane, the speckle decorrelation occurs. Second, we analyze the influence of wavelength diversity on the speckle decorrelation. As the wavelength diversity induces the axial shift of the reconstructed object, it affects speckle field propagation and also changes the imaging geometry of the speckle field. When the axial displacement is large enough, the speckle decorrelation occurs^44^. The speckle decorrelation by the angle and wavelength diversity is represented by

8 $$\begin{aligned} \begin{array}{l} {\mathop {\hbox {corr}}\nolimits } ({I_i},{I_j}) \approx \left| {{\mathop {\hbox {sinc}}\nolimits } \left( {\frac{{{w_e}{z_o}}}{{{\lambda _c}{z_s}{z_u}}}{x_{ij}}} \right) {\mathop {\hbox {sinc}}\nolimits } \left( {\frac{{{w_e}{z_o}}}{{{\lambda _c}{z_s}{z_u}}}{y_{ij}}} \right) } \right| ^2 \left| {{\mathop {\hbox {sinc}}\nolimits } \left( {\frac{{{w_e}^2{z_o}}}{{8{\lambda _c}^2{z_u}^2}}{\lambda _{ij}}} \right) } \right| ^2, \end{array} \end{aligned}$$

where $({x_{ij}},{y_{ij}},0)$ is ${\mathbf{{x}}_i} - {\mathbf{{x}}_j}$, $\lambda _{ij}$ is $\lambda _i-\lambda _j$, and $\lambda _c$ is the central wavelength of the light source. We assume that the correlation is determined by the multiplication of angle and wavelength diversity’s contribution. Detailed derivation of the equations in this section is described in Supplementary Material.

### Light source optimization

In the previous sections, we derived the theoretical models that quantify the resolution and the speckle contrast in partially coherent holographic displays. The theoretical models allow us to optimize light source characteristics for the best performance. The light source optimization aims to make the transfer function of holographic displays near diffraction limit, while minimizing the speckle contrast as much as possible. This optimization is represented by following problem.

9 $$\begin{aligned} \mathop {{\mathop {\hbox {minimize}}\nolimits } }\limits _{M,a} \sum \limits _{i = 1}^{{N_z}} {\left( {\left\| {{T_d}({\mathbf{{v}}}) - {\mathop {\hbox {abs}}\nolimits } \left( {{T_h}({\mathbf{{v}}};z_u^i)} \right) } \right\| ^2 + \gamma {C_s}(z_u^i)} \right) }, \end{aligned}$$

where $N_z$ is the sampling number of the reconstruction depths $z_u^i$, $T_d({\mathbf{v}})$ is the transfer function of the ideal display system, $\gamma$ is an optimization coefficient, and $C_s(z)$ is the speckle contrast at a reconstruction depth of *z*. Using a stochastic gradient descent method such as Adam^45^, we can find the optimal characteristics of light source. In this study, the optimization coefficient ($\gamma$) is set as 0.2, and the iteration number for the optimization is 1000. For the optimization, we sample the apodization by 1 μm interval of − 100 μm to 100 μm, SPD of 0.25 nm interval of 507–557 nm, and reconstruction depths 1 mm interval of 58–77 mm. The maximum frequency of the observation images is limited to 30 cycles per degree (cpd). The cpd unit represents the angular resolution of observed image in human visual system. Detailed information about the differentiation of the merit function in Eq. (9) and the optimization algorithm is included in “Method” section and Supplementary Material.

**Figure 2:**
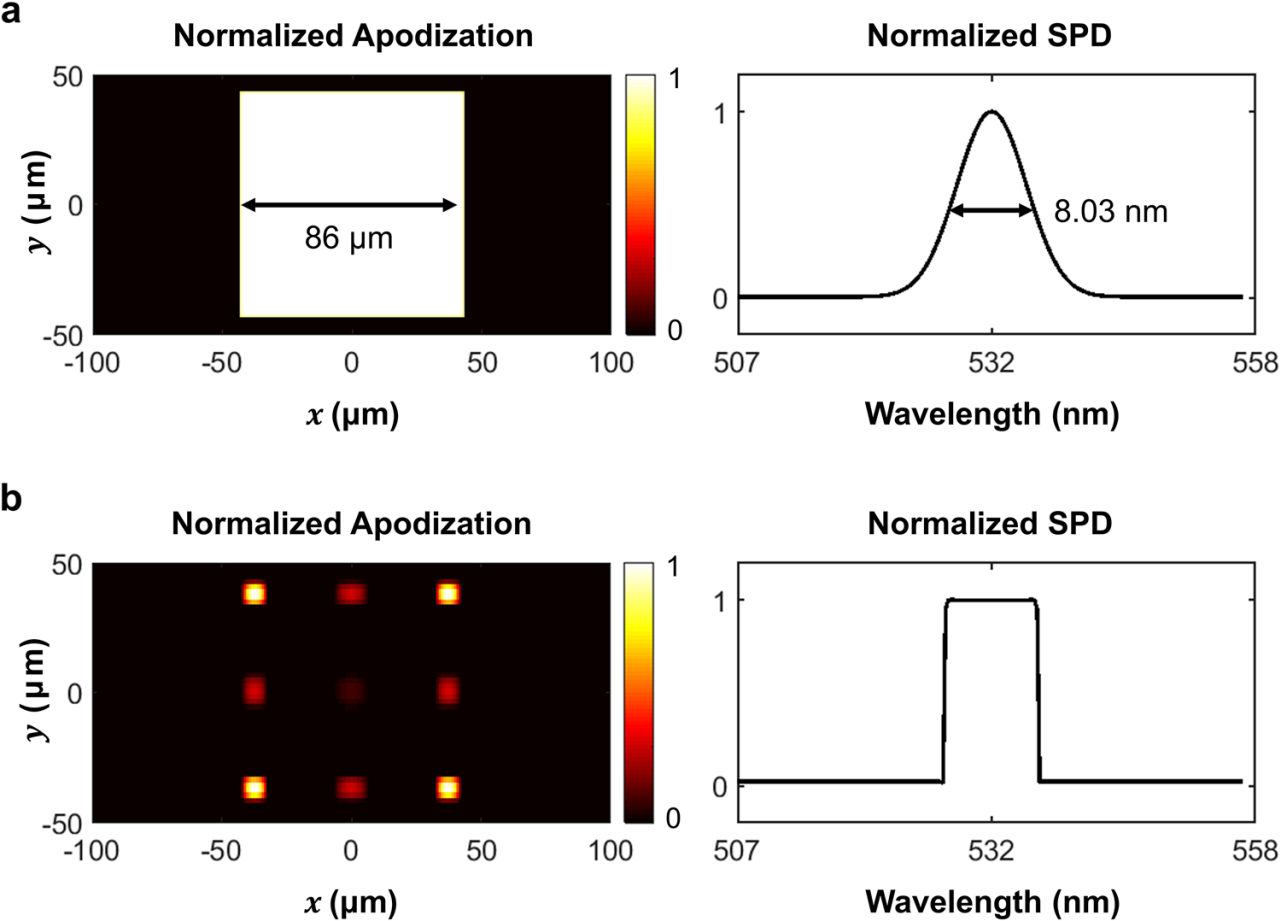
Optimization results of normalized apodization function and spectral power distribution (**a**) with predefined models and (**b**) without predefined models. With the predefined model, the optimal $\sigma _\lambda$ and $w_s$ are given by 3.41 nm (= 8.03 nm full width at half maximum, FWHM) and 86 μm, respectively.

The optimal light source is designed for a holographic display with following specifications. The focal length of the collimating lens ($z_s$) is 150 mm, and the distance between the SLM and the eye-lens ($z_o+z_u$) is 100 mm. The eye-lens’ pupil diameter ($w_e$) is 2.2 mm, and the eye-ball size ($z_e$) is 25 mm. Figure 2 illustrates the optimal SPD $M(\lambda )$ and the angle diversity *a*(*x*) for this holographic display. In Fig. 2a, we present optimization result with predefined models to ensure that the optimal light source can be manufactured. For the predefined models, we use the rectangular and Gaussian functions for the apodization and the SPD. Without the predefined models, the light source’s apodization and SPD are optimized as shown in Fig. 2b. It is noticeable that the optimal apodization is knobbly. The theoretical background for this result can be found in Eq. (8). The equation connotes that the speckle correlation function follows sinc functions that have several side-lobes. By the sinc functions, two speckle patterns are fully decorrelated only at specific conditions. It indicates that a square or a circular aperture for a light source is not the optimal aperture shape for speckle reduction. For example, a light source with a pinhole array aperture could be better candidate for speckle reduction where each pinhole is separated by the exact speckle decorrelation condition.

### Partially coherent holographic displays

**Figure 3:**
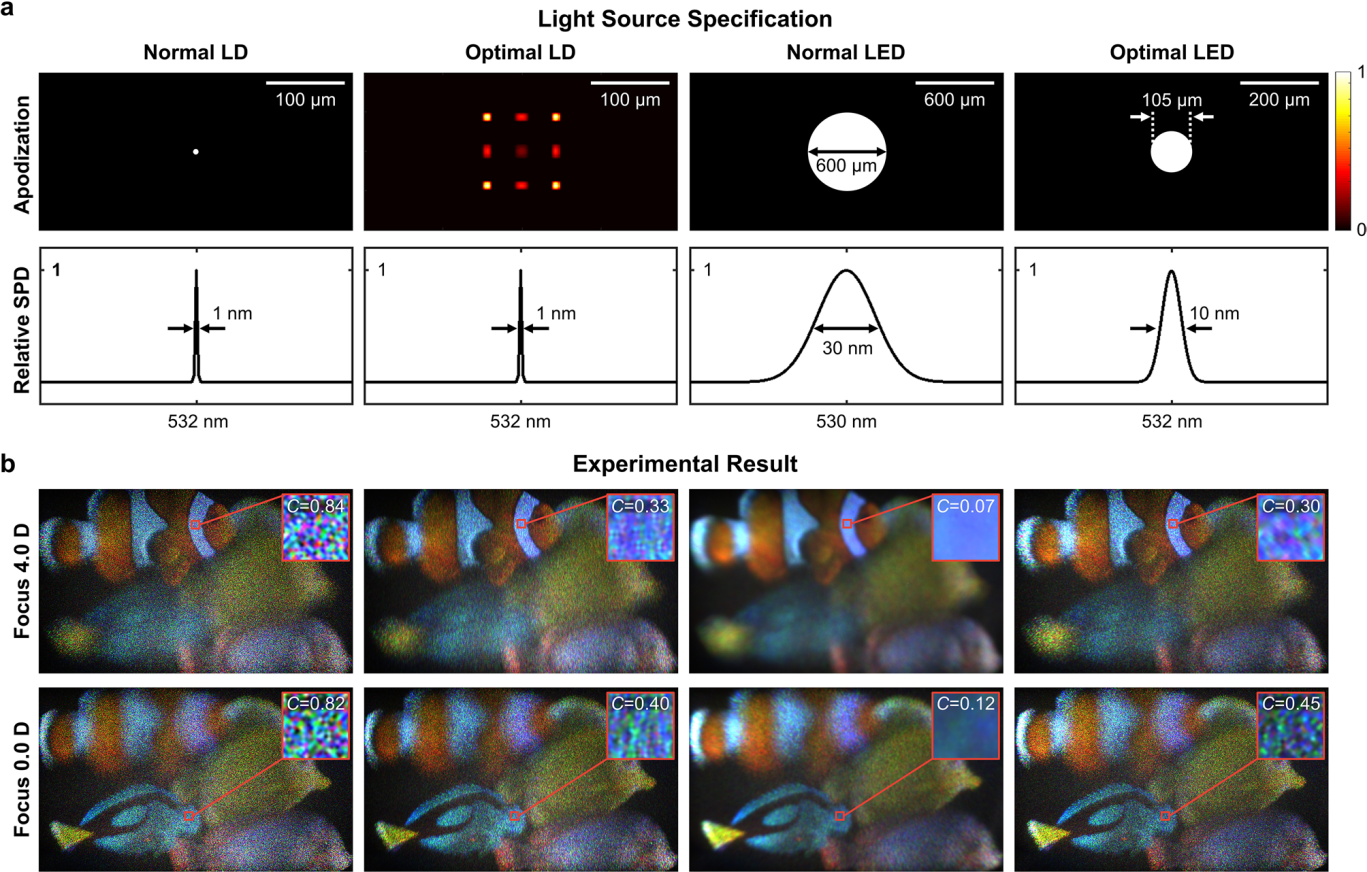
Experimental results to demonstrate holographic display performance using various light sources. We compare four light sources: a laser diode (LD), an optimally engineered LD (optimal LD), a light-emitting diode (LED), and an optimally engineered LED (optimal LED). (**a**) The apodization and relative SPD of each light source. (**b**) Holographic reconstruction results using corresponding light sources. The photographs present qualitative evaluation of resolution and SNR of holographic displays. The speckle contrast (*C*) is measured by cropping a small area of focused region. We present digitally synthesized RGB color images to explore how speckles appear in a full-color display. Each color channel image is captured using single light source demonstrated in the first row. Tropical fish (royalty-free 3D model from Turbosquid) is used for the experiment.

We implement a holographic display prototype to demonstrate the display performance according to the light source specifications. The prototype consists of a light source, an SLM, a 4f filtering system, and an eye-piece lens of 77 mm focal length. The optical configuration is identical with the illustration shown in Fig. 1. The central depth plane of the hologram is 33 mm from the SLM plane, which is separated by 67 mm from the eye-lens with an eye-piece. Light source candidates for the comparison are a laser diode (LD), an optimally engineered LD, a light-emitting diode (LED), and an optimally engineered LED. The apodization and the spectral distribution function of each light source are illustrated in Fig. 3a. The optimally engineered LD is designed by optimizing the apodization of an LD with the given SPD (1 nm FWHM). It can be reflected in the optimization algorithm by disregarding the update procedure of the SPD. On the other hand, both of the apodization and SPD are optimized for the optimally engineered LED. The optimally engineered LED has the most similar specifications to the optimal source shown in Fig. 2a.

An observation system to capture display results is a CCD camera (GS3-U3-91S6C-C, FLIR) with a c-mount lens (F/6D, 25 mm fixed focal length), which is similar with the human visual system. We estimate the exit-pupil diameter of the observation system at 2.2 mm, which is given by the speckle size of 6.05 μm. Figure 3b demonstrates volumetric reconstruction results of holographic displays using corresponding light sources. Each holographic display reconstructs a volumetric scene (tropical fish, royalty-free 3D model from Turbosquid) with 4.0 D depth range. Note that we digitally synthesize RGB color channel images to explore how speckles appear in a full-color display. Each color channel image is captured using single light source (i.e., green LD or LED). These results can not reflect the effect of the wavelength variation, and the speckle contrast and the resolution in a real use could be different. As demonstrated in the results, the optimal light sources enable more convincing display performance in terms of resolution, speckle contrast, and SNR. The optimal LD reduces the speckle contrast averagely by 44.0% (theoretical model: 47.5%), while minimizing the resolution degradation. The optimal LED also alleviates the noticeable resolution reduction of the normal LED. We measure the speckle contrast of each scene and compare with the theoretical model’s prediction. According to the theoretical model, the optimal LED averagely carries speckle reduction 51.5%, which is close to the experimental evaluation of 54.0%.

### Evaluation of theoretical model

To verify the theoretical model that predicts the resolution and the speckle contrast, we simulate partially coherent holographic displays illustrated in Fig. 1. In this simulation, we interpret a partially coherent light source as a synthesis of multiple coherent light sources. Each coherent light source generates a wavelet that illuminates a complex hologram with the different incident angles and wavelengths. The complex hologram reconstructs either a resolution chart or a white board at a specific depth. To evaluate the resolution and the speckle contrast, a single lens imaging system captures the reconstructed holographic images. By iterative loops, each wavelet’s holographic image is incoherently added up at the observation plane. The optical design parameters are identical with the prototype’s specifications. For example, the single lens imaging system has a circular exit-pupil $w_e$ of 2.2 mm diameter, and the distance of the exit-pupil from the observation plane $z_e$ is 25 mm.

**Figure 4:**
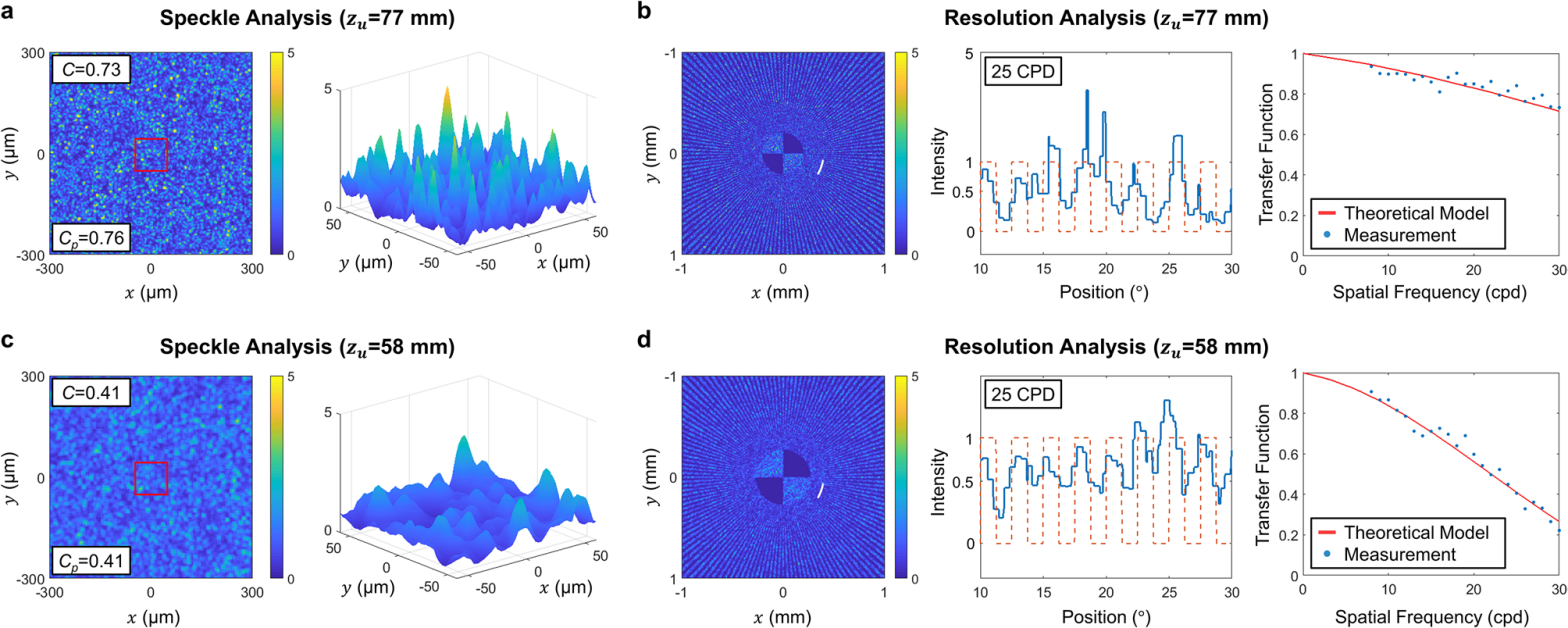
Simulation results to evaluate the theoretical models. Either a white board or a resolution chart is reconstructed at (**a**,**b**) 77 mm and (**c**,**d**) 58 mm. The simulation supposes the optimal light source with 86 μm by 86 μm aperture and Gaussian SPD with 3.41 nm standard deviation. (**a**,**c**) Illustration of the speckle patterns according to the light sources. The measured speckle contrast *C* is matched with the theoretical model’s prediction $C_p$. (**b**,**d**) The reconstructed resolution chart at the observation plane. The intensity information is magnified along the white lines, which corresponds to 25 cpd frequency. The transfer function is also calculated by Fourier transform of the intensity profile. The theoretical models for the speckle contrast ($C_p$) and the transfer function (red solid lines) are evaluated by the simulation results (*C* and blue dots).

Figure 4 demonstrates the simulation results that verify the prediction model for the resolution and the speckle contrast. The simulation results represent holographic reconstruction using the optimal light source shown in Fig. 2a. The light source’s aperture is 86 μm by 86 μm, central wavelength is 532 nm, and SPD follows Gaussian function with the standard deviation of 3.4 nm. The holographic images are reconstructed at three distances to the eye-lens: 58 mm, 67 mm, and 77 mm, which corresponds to the depth range considered in the light source optimization. Note that a holographic display using a coherent light source with the wavelength of 532 nm is a reference for the resolution analysis. As demonstrated in the results, the simulation fits well with the theoretical models for the resolution and the speckle contrast. Detailed procedures of the simulation are demonstrated in Supplementary Material with more results for the other reconstruction depths.

**Figure 5:**
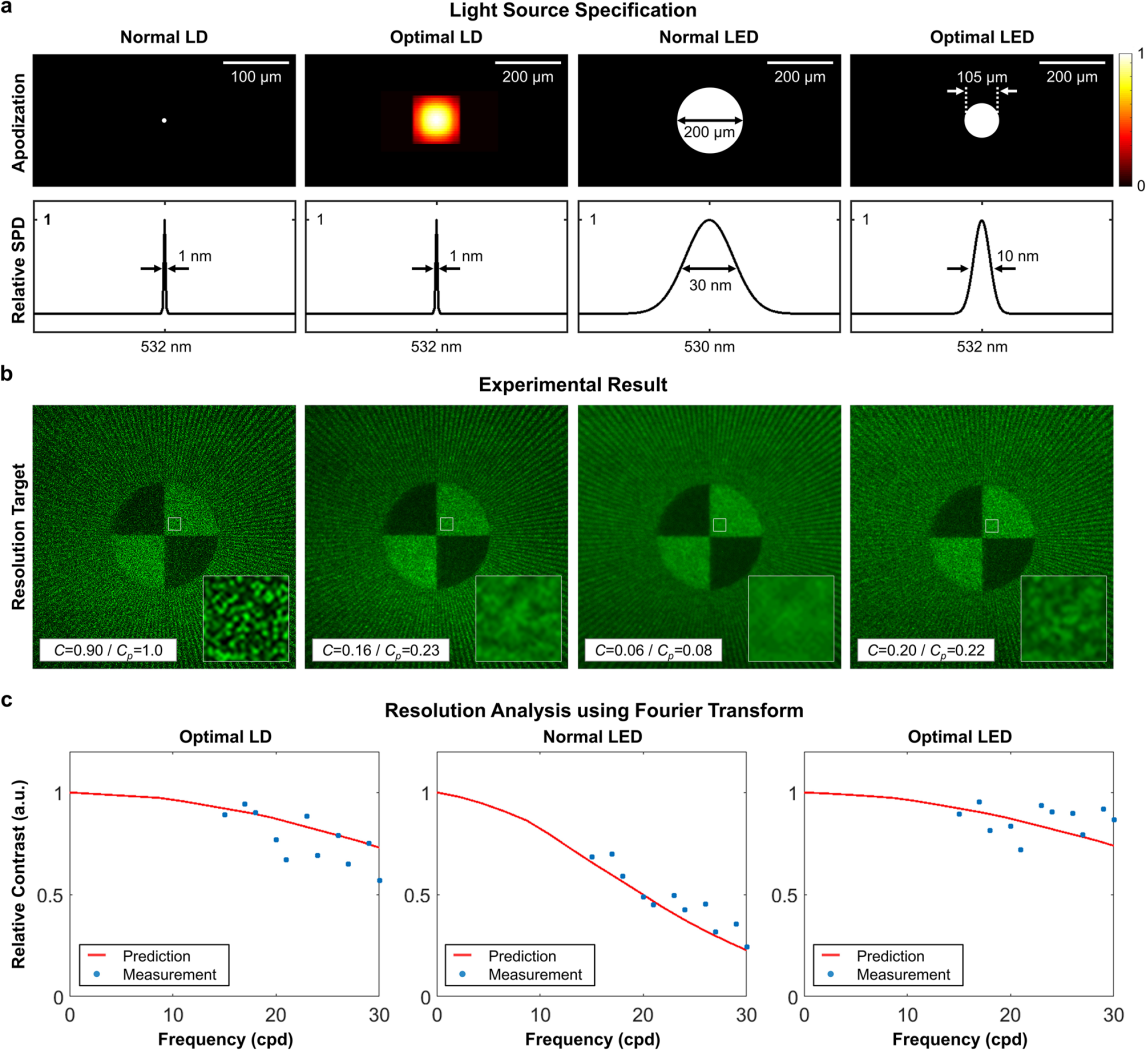
Experimental results to evaluate the resolution and the speckle contrast according to light source characteristics. (**a**) The apodization functions and the SPD of the light source candidates are illustrated. Note that the normal LED’s aperture is adjusted to 200 μm from 600 μm because 600 μm aperture carries too much resolution reduction that hinders accurate analysis. (**b**) Phase holograms generated by Wirtinger method^46^ reconstruct the resolution chart for resolution analysis. Using the experimental results, we estimate the speckle contrast as well as (**c**) the relative contrast function. Assuming the contrast function of the normal LD as the reference, we measure the relative contrast function (blue dots) and fit with the theoretical model (red solid lines).

We also demonstrate the experimental validation of the theoretical model in Fig. 5. In this experiment, the eye-piece lens is excluded as it usually carries several optical aberrations that might contaminate the resolution or speckle analysis. As the observation system should focus on a close distance without the eye-piece lens, a camera lens (AF Micro Nikon lens, 60 mm, F/8D) substitutes for the c-mount lens of 25 mm focal length. The exit-pupil diameter of the observation system is estimated at 8 mm, which is given by the speckle size of 3.99 μm. In accordance with the observation system modification, the distances of $z_e$ and $(z_o+z_u)$ are adjusted to 120 mm and 155 mm, respectively. The light source optimization is also repeated with the updated configuration. For the light source optimization, we sample the apodization by 3.85 μm interval of − 481 μm to 481 μm, and SPD 1 nm interval of 482–581 nm. The iteration number for the optimization is set as 2000. Note that the hologram depth $z_o$ is set as constant value of 65 mm. Other specifications not specified here are identical with the original configuration.

As illustrated in the first row of Fig. 5a, various light sources are materialized with different apodization and SPD. These light sources represent an LD, an optimally engineered LD, an LED, and an optimally engineered LED. For quantitative analysis of the resolution of holographic displays using those lights sources, we extract the Fourier coefficients of captured images (i.e., resolution chart) in Fig. 5b according to the frequency. To neutralize the resolution degradation involved by aberration of the optical elements, we derive the relative contrast function assuming the normal source’s result as the reference. Then, the relative contrast function is solely determined by the light source apodization function. We compare the experimental results with our theoretical model. As demonstrated in Fig. 5c, the theoretical model is well fitted with the experimental results. The speckle contrast of experimental results is also well matched with the theoretical model. The experimental results support the reliability of the theoretical model.

### Depth of field analysis

**Figure 6:**
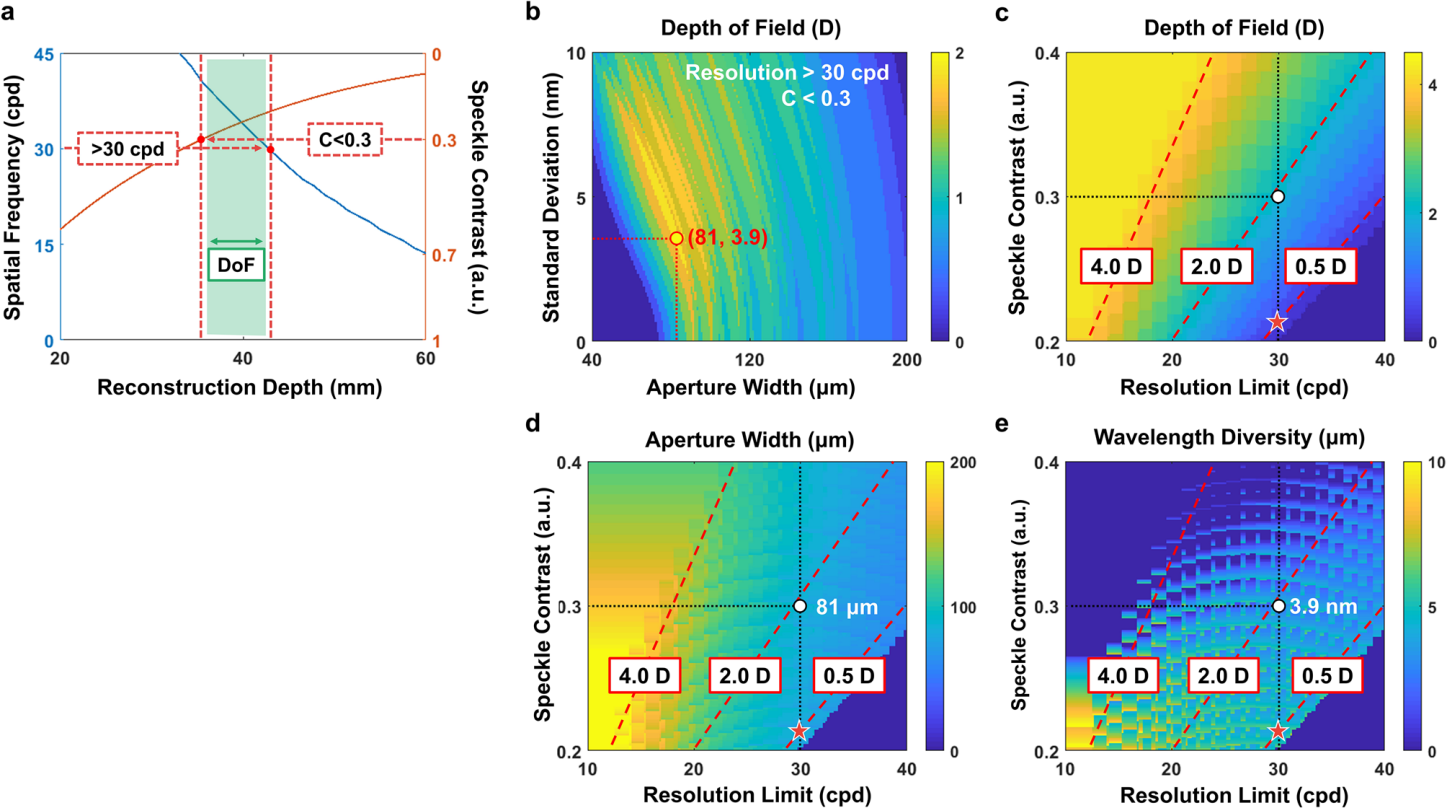
Demonstration of relationship among light source characteristics, resolution, speckle contrast, and depth of field in partially coherent holographic displays. (**a**) Illustration of the depth range where the target resolution (30 cpd) and speckle contrast (0.3) are achieved. (**b**) The depth of field according to the angle diversity and wavelength diversity of the light source. The red circle denotes the optimal light source specification ($w_s=81$ μm and $\sigma _\lambda =3.9$ nm). (**c**) The maximum depth of field according to the target resolution and speckle contrast when a light source of the optimal (**d**) angle diversity or (**e**) wavelength diversity is applied.

Along with the light source optimization, the theoretical models can be utilized to establish a criterion for depth of field evaluation of partially coherent holographic displays. As described in Eqs. (2) and (7)–(8), the reconstruction depth of the object ($z_o$) affects the resolution and the speckle contrast of holographic images. The correlation of the three terms connotes that the depth range is limited where high resolution images with low speckle contrast can be reconstructed. If we assume that there are target specifications for the resolution and the speckle contrast, each required specification determines appropriate reconstruction depth range. As illustrated in Fig. 6a, the overlapped region is specified where reconstructed images satisfy the target resolution (> 30 cpd) and the speckle contrast (< 0.3). We refer to the overlapped region as the depth of field in partially coherent holographic displays. For this figure, the observation system is set as a human visual system to analyze the depth range in a practical use. The eye-lens has a circular exit-pupil with 4 mm diameter, and the eye-ball size is 25 mm.

In Fig. 6b, we explore the depth of field in partially coherent holographic displays according to angle ($w_s$) and wavelength ($\sigma _\lambda$) diversity of the light source. Accumulating these data according to the target resolution and speckle contrast, we establish a light source library to investigate the relationship among resolution, speckle contrast, and depth of field. Figure 6c illustrates the maximum depth of field that we can achieve for the target resolution and speckle contrast. Figure 6d,e show the corresponding light source specifications to achieve the maximum depth of field. Using this light source library, we can optimize the light source characteristics for a specific purpose. Although the three figures are sorted to guide for the depth of field optimization, those figures are also applicable for optimization of other specifications. For example, we can minimize the speckle contrast while satisfying requirements of the resolution (30 cpd) and the depth of field (0.5 D). It can be done by finding an intersection point between the contour and vertical lines on the Fig. 6c. The intersection point is denoted by a red star in the figure. The optimal specifications of the light source are specified at the same point in Fig. 6d,e.

Figure 6c–e show that the sacrifice of depth of field is inevitable for considerable speckle reduction. The limited depth of field is a critical bottleneck of partially coherent holographic displays. Inspired by previous works using a tunable-focus lens for gaze-contingent displays^47^ or tomographic displays^13,48^, we can consider an adaptive focus holographic display system. According to the gaze direction, the depth information of the scene is adjusted to reconstruct gazed objects mostly within the limited depth of field. The adjustment of the depth information is compensated by the tunable-focus lens to provide accurate accommodation. For example, if we increase the tunable-focus lens power by 2.0 diopters (D), the eye-lens optical power is decreased by 2.0 D to observe clear holographic images at a central depth plane (CDP). The decrease of the eye-lens optical power is recognized as observation of a closer object. In Supplementary Material, we discuss more details of the adaptive focus holographic display with experimental results.

### Limitation of theoretical models and light source optimization

Our theoretical models for speckle and resolution analysis have some assumption or approximation. First, we suppose an on-axis imaging geometry where the magnification variation of the imaging geometry is negligible. Second, the SPD of the light source is sufficiently narrow so that the reconstruction depth variation is negligible. Third, we approximate 2D speckle correlation model using multiplication of independent 1D speckle correlation models. Although these assumption enables differentiable quantitative models, the models could be unreliable in a specific condition where the assumptions are inappropriate. For instance, if the field of view or the SPD is wide, the resolution and the speckle contrast are reduced more than expectation. The third assumption is for simplified derivation of the gradient function in Eq. (9). As the shape of speckle pattern is a circle rather than a square, the precise 2D speckle correlation model is Bessel function defined in the cylindrical coordinates. Thus, there is some error in our representation of the speckle correlation function using the multiplication of two independent and orthogonal sinc functions.

Although we formulated the optimization problem of the light source in Eq. (9), it is not intuitive how to determine the optimization coefficient $\gamma$. In this study, we empirically determined the optimization coefficient as 0.2, which gave the most convincing display performance. However, it could be a subjective decision to find an agreed point between the speckle contrast and the resolution. If someone believes the speckle reduction is more important, the optimization coefficient should be adjusted to a higher constant. As the optimization result is sensitive to the optimization coefficient $\gamma$, it is important to find an appropriate value. For appropriate determination of the optimization coefficient, we might need a user study to evaluate the viewing experience of partially coherent holographic displays. The goal of this user study would be evaluation of perceived resolution and contrast of holographic images, and visual fatigue involved by coherent illumination. Supplementary Material investigates the contribution of $\gamma$ on the light source optimization with qualitative analysis.

## Discussion

We have investigated the theoretical background of partially coherent holographic displays. The analytical model was established to quantify the speckle contrast and the resolution according to the optical characteristics of the partially coherent light source. Using the analytical model, we verified that partially coherent holographic displays suffer from the trade-off among resolution and speckle contrast. To alleviate the trade-off problem, we optimized the angle and wavelength diversity of the light source to achieve optimal viewing experience. Simulation and experimental results were demonstrated to validate our analytical model for partially coherent holographic displays. We also introduced the criterion to determine the depth of field where holographic images are reconstructed with a suitable level of resolution and speckle contrast. Despite the light source optimization, we noticed that the sacrifice of the depth of field or resolution is inevitable for considerable speckle reduction. To alleviate the trade-off, we conceived partially coherent holographic displays using adaptive focus method. Additional benchtop prototype demonstrated the experimental results that validate the feasibility of the proposed method. Finally, we have discussed approximations and limitations in the theoretical models and light source optimization. We believe that our work could inspire a new holographic display system with less speckle contrast with improved SNR and secured resolution.

## Method

### Algorithm for light source optimization

To solve the optimization problem given by Eq. (9), we should find the optimal parameters for the apodization function, $p_s({\mathbf{x}})$ and the SPD, $M(\lambda )$ of the light source. We approach this problem with two-step updates: We firstly update $p_s({\mathbf{x}})$ assuming $M(\lambda )$ is constant, and secondly update $M(\lambda )$ assuming $p_s({\mathbf{x}})$ is constant. We employ vectorization form of the apodization function and the SPD to find the optimal parameter set with the full degree of freedom. For the optimization with the predefined models, we represent the SPD using Gaussian function where the standard deviation is a parameter to be optimized. The apodization function of the light source is also set as rectangular function where the aperture width is the optimization parameter. In the optimization using the predefined models, we set the optimization coefficient $\gamma$ as 0.2. To update the standard deviation $\sigma _\lambda$ of the SPD, we employ a steepest gradient descent method with coefficient of $2\times 10^{-17}$. On the other hand, we use Adam^45^ for updating the aperture width $w_s$ to avoid the local minima. The optimization algorithm’s constants are $\alpha =10^{-6}$, $\beta _1=$0.9, $\beta _2=$0.999, and $\epsilon =10^{-8}$. Initial conditions for $\sigma _\lambda$ and $w_s$ are given by 4.25 nm and 100 μm, respectively.

For the optimization without the predefined model, update parameters are $M(\lambda )$ and $a({\mathbf{x}})$ instead of $\sigma _\lambda$ and $w_s$. In this case, we also apply Adam for updating $M(\lambda )$ because the solution space is no longer guaranteed as convex. The optimization constants for Adam algorithm are $\alpha =5\times 10^{-7}$, $\beta _1=$0.9, $\beta _2=$0.999, and $\epsilon =10^{-8}$. The initial conditions for $M(\lambda )$ and $a({\mathbf{x}})$ are found by $\sigma _\lambda$ of 4.25 nm and $w_s$ of 100 μm. Note that we applied additional constraints on the apodization and SPD functions to minimize a sampling issue. Both functions are forced to be symmetric, and have some marginal spaces to secure a zero-padding area. The marginal space size is determined by the maximum aperture size that enables the 30 cpd resolution. For example, the maximum aperture size is set as 120.5 μm for the optimization shown in Fig. 2b. To validate the optimization algorithm, we demonstrate the convergence graph in Supplementary Material. We confirm that the introduced iterative algorithm converges to the optimal point.

### Light source implementation

The various types of the laser diode in Figs. 5 and 6 are materialized by engineering the angle diversity of a laser diode (532 nm, CPS532-C2, Thorlabs). We exploit a DMD system (DLP9500, Texas Instrument) to increase the angle diversity via temporal multiplexing of spatially distorted wavelets. The DMD displays a sequence of binary holograms with temporal multiplexing, which can materialize an optically equivalent illumination to the partially coherent source with angle diversity. For example, the DMD can reconstruct pinholes sequentially, which corresponds to an array of mutually incoherent light sources. Each sequential DMD pattern reconstructs a pinhole at different lateral position in Fourier plane. Lateral positions correspond to illumination angles for an SLM (JD8714, Jasper display) where hologram of object is displayed. When the SLM is illuminated by sequential beams in a fast frame rate, different speckle patterns are temporally fused. More details of the implementation including specifications are shown in Supplementary Material. Note that the optimal apodization function is found with the given SPD (< 1 nm FWHM). It was reflected in the optimization algorithm by disregarding the update procedure of the SPD.

The various types of the LED sources used in Figs. 5 and 6 are implemented by a combination of a fiber-coupled LED module (530 nm, M530F2, Thorlabs), multimode fiber patch cables with different core diameter (105 μm, 200 μm, and 600 μm, SMA to SMA series, Thorlabs), and a bandpass filter (FWHM 10 nm, FL532-10, Thorlabs). For example, the optimal light source in Fig. 5 is implemented by the fiber-coupled LED module with the 105 μm multimode cable, and the bandpass filter. The bandpass filter is located between the collimating lens for the LED and the SLM. Without the bandpass filter, the fiber-coupled LED module has the spectral bandwidth of 30 nm FWHM. We can also use other light sources to investigate the contribution of the SPD, such as superluminescent LEDs that have relatively narrower FWHM less than 10 nm. Note that the optimal apodization and SPD functions are found with the predefined models. The predefined models were a square aperture for the apodization and a Gaussian function for the SPD. The optimal source for Fig. 5 is supposed to have 109.2 nm aperture and 9.5 nm FWHM.

### Hologram generation

Binary holograms for the DMD ($1920 \times 1080$ pixels, 10.8 μm) to engineer the angle diversity are generated by following analog hologram principle. Analog holograms (real numbers) are formed by two wave interference^49^. One is illumination for the DMD, and the other is what we want to reconstruct. The hologram is converted to binary hologram by hardclipping, which is displayed by the DMD. Unwanted terms from hologram (ambiguity, conjugate, high-order) are optically filtered at Fourier plane. To alleviate the signal noises, we placed an additional physical pinhole with a radius of 250 μm. Binary hologram sequence of the DMD is designed to generate sequential illumination beams for SLM, which temporally updates speckle pattern. The temporal multiplexing of the binary hologram sequence aims to reconstruct the optimal apodization function at the Fourier plane. The apodization is materialized by temporal multiplexing of 150 binary holograms reconstructed by the DMD.

We generate phase-only holograms for the SLM ($3840 \times 2160$ pixels, 3.74 μm) using two different methods for speckle comparison. First, we apply a primitive method that simply replaces the amplitude of complex holograms as 1. To derive the complex holograms, the target complex amplitude inversely propagates to the SLM plane. The primitive method is employed for the experiment in Fig. 6, where the holograms reconstruct volumetric scenes. Second, we optimize phase-only holograms following the method called Wirtinger holography^46^. We apply 100 iterations for the hologram optimization. The Wirtinger hologram is employed for the experiment in Fig. 5, where the hologram reconstructs the flat scene. For the all experiment, the zero-order signal from the SLM is blocked by a 4f filtering system. Note that we need to tilt the SLM to neutralize the slanted illumination of the engineered light source. The resolution of the target complex amplitude is set as $2160 \times 2160$.

## Supplementary information

Supplementary Information.

## Data Availability

The data that support the plots within this paper and other findings of this study are available from the corresponding author upon reasonable request.
