# Supplementary material for: Light source optimization for partially coherent holographic displays with consideration of speckle contrast, resolution, and depth of field
Source: Sci Rep. 2020 Nov 2;10:18832. doi: 10.1038/s41598-020-75947-0 (PMC7606540; doi:10.1038/s41598-020-75947-0)
Supplement: Supplementary file 1 — Supplementary Information. [file 41598_2020_75947_MOESM1_ESM.pdf]

Supplementary Information
Light Source Optimization for Partially Coherent Holographic Displays with Consideration of Speckle Contrast, Resolution, and Depth of Field

Seungjae Lee[†], Dongyeon Kim[†], Seung-Woo Nam , Byounghyo Lee , Jaebum Cho , and Byoungho Lee[*]

School of Electrical and Computer Engineering, Seoul National University, Gwanak-Gu Gwanakro 1, Seoul, 08826, Republic of Korea
[*]byoungho@snu.ac.kr
[†]These authors contributed equally to this paper.

Supplementary Note 1: Derivation of Equations

Resolution

When we suppose the object plane $o(\mathbf{a})$ at $z = z_o$ and the SLM plane at $z = 0$, complex amplitude of the ideal SLM $s(\mathbf{x})$ is given by

$$s(\mathbf{x}) = \int h_{\lambda_o}(\mathbf{x}-\mathbf{a};-z_o)o(\mathbf{a})d\mathbf{a}, \tag{S1}$$

where $h_{\lambda_o}(\mathbf{x})$ is the impulse response function of free space when the wavelength of the object illumination is λ_o, and $o(\mathbf{a})$ is complex amplitude of the object. If the SLM is illuminated by a plane wave whose complex amplitude is $\exp[j2\pi(\mathbf{v}_s \cdot \mathbf{x})]$ at z=0, the SLM converts the complex amplitude of the incident wave into

$$u(\mathbf{x}) = s(\mathbf{x})\exp(j2\pi(\mathbf{v}_s \cdot \mathbf{x})). \tag{S2}$$

The Fourier transform of the complex amplitude at the reconstructed object plane $z = \tilde{z}_o$ is

$$\begin{aligned} G(\mathbf{v}) &= H_{\lambda_s}(\mathbf{v};\tilde{z}_o)U(\mathbf{v}) \\ &= H_{\lambda_s}(\mathbf{v};\tilde{z}_o)H_{\lambda_o}(\mathbf{v}-\mathbf{v}_s;-z_o)O(\mathbf{v}-\mathbf{v}_s). \end{aligned} \tag{S3}$$

If we apply Fresnel approximation and substitute $\tilde{z}_o = \lambda_o z_o/\lambda_s$ into Eq. S3, $G(\mathbf{v})$ is given by

$$G(\mathbf{v}) \approx C(\mathbf{v}_s)\exp\left(-j2\pi\lambda_o z_o\left(\mathbf{v}_s \cdot \mathbf{v}\right)\right)O(\mathbf{v}-\mathbf{v}_s), \tag{S4}$$

where $C(\mathbf{v}_s)$ is a constant given by $\exp\left(j\pi z_o\lambda_o\left(v_{sx}^2+v_{sy}^2\right)\right)$. Accordingly, the complex amplitude at $z = \lambda_o z_o/\lambda_s$ is

$$g(\mathbf{a};-\lambda_o z_o/\lambda_s) = c(\mathbf{v}_s)o(\mathbf{a}-\lambda_o z_o\mathbf{v}_s)\exp\left(j2\pi(\mathbf{v}_s \cdot \mathbf{a})\right). \tag{S5}$$

This equation indicates that the reconstructed object is shifted by $\left(\lambda_o z_o v_{sx}, \lambda_o z_o v_{sy}, \frac{\lambda_o-\lambda_s}{\lambda_s}z_o\right)$ while illuminated by a plane wave at angles $\theta_x = \sin^{-1}\lambda_s v_{sx}$ and $\theta_y = \sin^{-1}\lambda_s v_{sy}$.

As we suppose that wavelets are mutually incoherent, an user observes the intensity sum of the laterally and axially shifted objects. To analyze how shifted objects are synthesized at the observation plane, we first add up the intensity of laterally shifted objects, which are reconstructed by wavelets with a wavelength of $\tilde{\lambda}_s$. The sum of the objects at the depth of $z = \lambda_o z_o/\tilde{\lambda}_s$ is the convolution of the rectangular function and the original object because the angle diversity is determined by the light source square aperture. Thus, the multiplication of a 2D sinc function and the Fourier transform of the object image corresponds to the Fourier transform of the intensity sum I_A, which is given by

$$\begin{aligned} I_A(\mathbf{v};\tilde{\lambda}_s) &= A(\mathbf{v})I_o(\mathbf{v}) \\ &= \operatorname{sinc}\left(\frac{w_s}{z_s}\frac{\lambda_o}{\tilde{\lambda}_s}z_o v_x\right)\operatorname{sinc}\left(\frac{w_s}{z_s}\frac{\lambda_o}{\tilde{\lambda}_s}z_o v_y\right)I_o(\mathbf{v}), \end{aligned} \tag{S6}$$

where $A(\mathbf{v})$ is the rectangle function determined by the light source aperture and I_o is the Fourier transform of the object image. v_x and v_y are x and y components of the wave vector $\mathbf{v}$.

Second, the intensity sum at the the depth of $z = \lambda_o z_o/\tilde{\lambda}_s$ is transferred to the observation plane. When the user focuses on the reference depth of $z = z_o$, the amplitude transfer function H of the observation system is known[1] as

$$\begin{aligned} H_{\tilde{\lambda}_s}(\mathbf{v}) &= P\left(\tilde{\lambda}_s z_e\mathbf{v}\right)\exp\left(-jkW(\tilde{\lambda}_s z_e\mathbf{v})\right), \\ W(\tilde{\lambda}_s z_e\mathbf{v}) &= -\frac{\tilde{\lambda}_s^2 z_e^2}{2}\left(\frac{1}{z_u}-\frac{1}{\tilde{z}_u}\right)\left(v_x^2+v_y^2\right), \end{aligned} \tag{S7}$$

where P is the Fourier transform of the pupil function, and W is an effective phase-length error given by a focus error of the observation system. z_u and $\tilde{z}_u$ are the distance between the eye-lens and the reference depth and the object depth. We note that the optical transfer function T of the incoherent imaging system is given by the normalized autocorrelation of the amplitude transfer function[1]. Using the optical transfer function, we calculate the Fourier transform of the observation image R_A as

$$\begin{aligned} R_A(\frac{\tilde{z}_u}{z_e}\mathbf{v};\tilde{\lambda}_s) &= T(\mathbf{v};\tilde{\lambda}_s)\tilde{I}_A(\mathbf{v};\tilde{\lambda}_s) \\ &= T(\mathbf{v};\tilde{\lambda}_s)A(\mathbf{v};\tilde{\lambda}_s)I_o(\mathbf{v}), \end{aligned} \tag{S8}$$

where the imaging geometry of the eye-lens magnifies the lateral shift of the reconstructed object at the observation plane.

Speckle Contrast

To derive the speckle contrast equation, we start from the definition. The speckle contrast is defined as m_{I_s}/σ_{I_s} that is the ratio of the intensity average to the standard deviation. First, the intensity average of the sum of speckle patterns is given by

$$\begin{aligned}
\mathrm{E}(I_s) &= \mathrm{E}\left(\sum_i c(\mathbf{t}_i) I_i \Delta\mathbf{t}_i\right) \\
&= \sum_i \mathrm{E}\left(I_i\right) c(\mathbf{t}_i)\Delta\mathbf{t}_i \\
&= m_I \sum_i c(\mathbf{t}_i)\Delta\mathbf{t}_i,
\end{aligned} \tag{S9}$$

where each speckle pattern is the fully developed speckle that has the identical average intensity m_I as well as the standard deviation σ_I. Next step is to derive the standard deviation. The standard deviation can be calculated if we know the average of the intensity square, which is given by

$$\begin{aligned}
\mathrm{E}({I_s}^2) &= \mathrm{E}\left(\left(\sum_i c(\mathbf{t}_i) I_i \Delta\mathbf{t}_i\right)^2\right) \\
&= \mathrm{E}\left(\left(\sum_i c(\mathbf{t}_i) I_i \Delta\mathbf{t}_i\right)\left(\sum_j c(\mathbf{t}_j) I_j \Delta\mathbf{t}_j\right)\right) \\
&= \mathrm{E}\left(\sum_j \sum_i I_i I_j c(\mathbf{t}_i) c(\mathbf{t}_j)\Delta\mathbf{t}_i\Delta\mathbf{t}_j\right) \\
&= \sum_j \sum_i \mathrm{E}\left(I_i I_j\right) c(\mathbf{t}_i) c(\mathbf{t}_j)\Delta\mathbf{t}_i\Delta\mathbf{t}_j.
\end{aligned} \tag{S10}$$

We substitute the covariance $cov(I_i, I_j)$ to the equation, which is defined as

$$\mathrm{E}\left(I_i I_j\right) = \mathrm{E}(I_i)\mathrm{E}(I_j) + \mathrm{cov}(I_i, I_j). \tag{S11}$$

The equation is organized to

$$\mathrm{E}({I_s}^2) = \mathrm{E}(I_s)^2 + \sum_j \sum_i \mathrm{cov}(I_i, I_j) c(\mathbf{t}_i) c(\mathbf{t}_j)\Delta\mathbf{t}_i\Delta\mathbf{t}_j. \tag{S12}$$

Finally, we can derive the variance of the intensity as follows.

$$\begin{aligned}
\mathrm{Var}(I_s) &= \mathrm{E}({I_s}^2) - (\mathrm{E}(I_s))^2 \\
&= \sum_j \sum_i \mathrm{cov}(I_i, I_j) c(\mathbf{t}_i) c(\mathbf{t}_j)\Delta\mathbf{t}_i\Delta\mathbf{t}_j.
\end{aligned} \tag{S13}$$

We remember that the speckle contrast equation is given by the ratio of the standard deviation and the average as follows.

$$C = \frac{\sigma(I_s)}{\mathrm{E}(I_s)} = \frac{\sigma(I_s)}{m_I \sum_i c(\mathbf{t}_i)\Delta\mathbf{t}_i} = \frac{\sigma(I_s)}{\sigma_I \sum_i c(\mathbf{t}_i)\Delta\mathbf{t}_i}. \tag{S14}$$

By substituting the Eq. S9 and Eq. S13 into the above equation, we get

$$\begin{aligned}
C^2 &= \frac{\sum_j \sum_i \mathrm{cov}(I_i, I_j) c(\mathbf{t}_i) c(\mathbf{t}_j)\Delta\mathbf{t}_i\Delta\mathbf{t}_j}{{\sigma_I}^2 \left(\sum_i c(\mathbf{t}_i)\Delta\mathbf{t}_i\right)^2} \\
&= \frac{1}{\left(\sum_i c(\mathbf{t}_i)\Delta\mathbf{t}_i\right)^2} \sum_j \sum_i \frac{\mathrm{cov}(I_i, I_j)}{\sigma(I_i)\sigma(I_j)} c(\mathbf{t}_i) c(\mathbf{t}_j)\Delta\mathbf{t}_i\Delta\mathbf{t}_j \\
&= \frac{\sum_j \sum_i \mathrm{corr}(I_i, I_j) c(\mathbf{t}_i) c(\mathbf{t}_j)\Delta\mathbf{t}_i\Delta\mathbf{t}_j}{\left(\sum_i c(\mathbf{t}_i)\Delta\mathbf{t}_i\right)^2}.
\end{aligned} \tag{S15}$$

Note that we employ the relationship between the correlation and covariance.

Supplementary Note 2: Light Source Optimization

To derive the gradient of the merit function, we first conceive a system matrix for the holographic displays. In other words, we need to convert the Eq. 9 into the form described by

$$\underset{\mathbf{q}}{\text{minimize}}\, \|\mathbf{y}-|\mathbf{S}f(\mathbf{q})|\|^2+\textstyle\sum_i^{N_z}\gamma C_s\left(g(\mathbf{q})\right), \tag{S16}$$

where $\mathbf{y}_i$ is the vectorized target transfer function according to the reconstruction depth and the spatial frequency. The vector length of $\mathbf{y}_i$ is $N_a N_z$. $\mathbf{S}$ is the system matrix of the holographic display where the matrix dimension is $N_a N_z \times N_q$. $\mathbf{q}$ is the vectorized parameters whose length is N_q. C_s^i is the speckle contrast function at the depth z_i. Note that the function f and g are system modeling functions that connect the system matrix and the parameters. For instance, the rectangular function could be considered for f to return vectorized apodization $p_s(\mathbf{x})$. Note that there are two different forms of the merit function as we optimize two different parameter sets separately. In single iteration, two updates are conducted for the apodization (aperture width) and the spectral power distribution (standard deviation of the spectrum).

Basically, the system matrix is designed for the integration given by Eq. 2. The integration along the wavelength can be expressed by matrix multiplication of the system matrix and the vectorization of corresponding parameters. Note that the matrix elements are generated by considering three variables of spatial frequency, wavelength, and reconstruction depth. For updating apodization, the system matrix corresponds to the multiplication of $M(\lambda_s)$ and $T(\mathbf{v};\lambda_s)$. The system matrix is multiplied with the Fourier transform of the apodization which can also be described by matrix multiplication. Thus, $f(\mathbf{q})$ is given by

$$f(\mathbf{q})=\left|\mathbf{F}\frac{\mathbf{q}}{\sum_i q_i}\right|, \tag{S17}$$

where $\mathbf{F}$ is the Fourier transform matrix whose dimension is $N_a \times N_a$. $g(\mathbf{q})$ is simply given by $\mathbf{q}/\sum_i q_i$. $\mathbf{q}$ is the vectorization form of the apodization. For updating spectral power distribution, the system matrix corresponds to the multiplication of $T(\mathbf{v};\lambda_s)$ and $A(\mathbf{v};\lambda_s)$. The system matrix is multiplied with the spectral power distribution $M(\lambda)$. At this time, $f(\mathbf{q})$ is given by

$$f(\mathbf{q})=\frac{\mathbf{q}}{\sum_i q_i}, \tag{S18}$$

which is identical with $g(\mathbf{q})$. $\mathbf{q}$ is the vectorization form of spectral power distribution.

Finally, the gradient of Eq. S16 is given by

$$2\mathbf{S}^{\dagger}\left(\mathbf{S}f(\mathbf{q})-\mathbf{y}\circ\frac{\mathbf{S}f(\mathbf{q})}{|\mathbf{S}f(\mathbf{q})|}\right)\frac{\partial f(\mathbf{q})}{\partial\mathbf{q}}+0.5\gamma C_0\textstyle\sum_i^{N_z}\left(\left(\mathbf{Cr}_i+\mathbf{Cr}_i{}^{\dagger}\right)g(\mathbf{q})\right)\frac{\partial g(\mathbf{q})}{\partial\mathbf{q}} \tag{S19}$$

where $\mathbf{S}^{\dagger}$ is the conjugate transpose of $\mathbf{S}$, and $\circ$ indicates the Hadamard product. Note that the division is also element-wise product of two vectors. $\mathbf{Cr}_i$ is the correlation matrix for the depth of z_i. C_0 is the speckle reduction term by the other diversity contribution. By substituting the equations for f and g into the gradient equation, we can derive the gradient of the merit function. Note that if we use a specific model for the apodization or spectral power distribution, the additional gradient calculation is supplemented to these equations. To confirm that our optimization algorithm converges to the optimal point, we demonstrate the convergence graph in Fig. S1. As we can see in the figure, the optimization algorithm converges to the global minimum point without trapping at local minima. We also notice that the merit function could be more reduced if we optimize without the predefined model.

Optimization Coefficient

In Fig. S2, we investigate the contribution of γ on the light source optimization. If we choose a gamma, we can find an optimal light source with a specific angle and wavelength diversity. From this point, we recalculate the resolution and the speckle contrast curve according to reconstruction depth, and evaluate the depth of field. For the evaluation, the target resolution is set as 30cpd because it is known as the resolution limit of normal vision. Then, the depth of field is evaluated according to the target speckle contrast. As shown in the figure, the optimization coefficient of 0.2 guarantees the reliable performance for the 2.2 mm exit-pupil observation system. On the other hand, the optimization coefficient of 0.6 could be a better option for a practical use, where the exit-pupil size is 4 mm.

Supplementary Note 3: Holographic Reconstruction Simulation

Pipeline

As described in manuscript, we suppose a partially coherent light source as a synthesis of multiple coherent light sources with the different incident angles and wavelengths. In the simulation, each coherent light source is collimated so that a plane

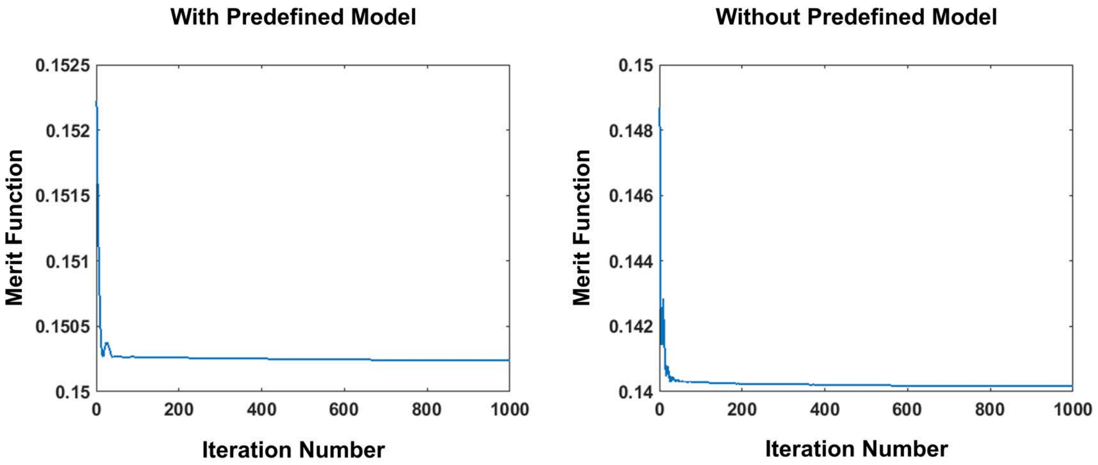

Supplementary Figure S1. Illustration of the convergence graph of the light source optimization with or without the predefined model.

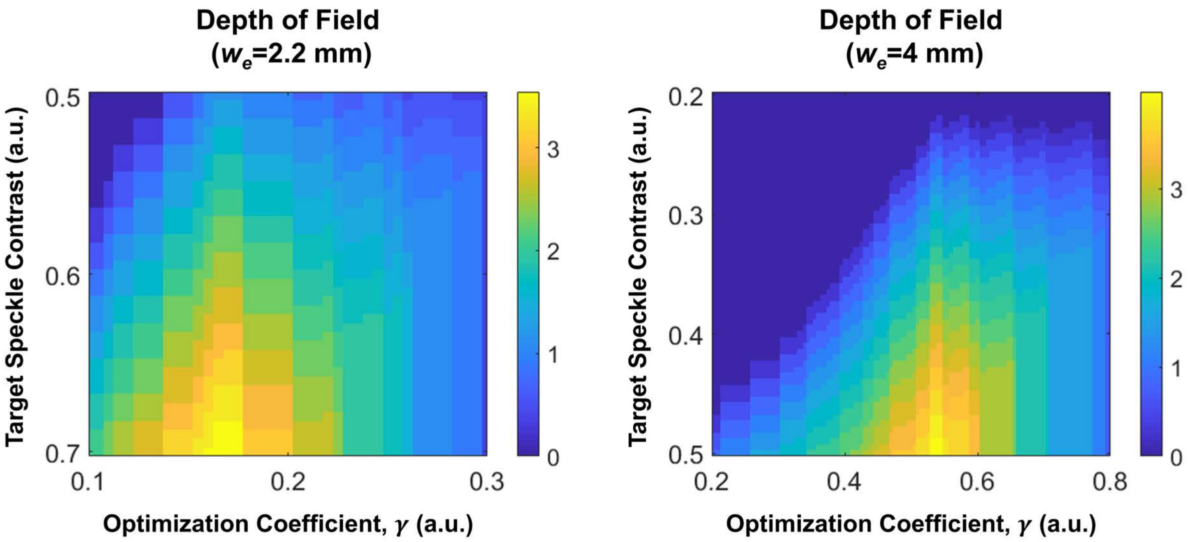

Supplementary Figure S2. Evaluation of the optimization coefficient γ by the depth of field.

wavefront with a specific angle and wavelength is generated. These wavelets are introduced to an SLM where the complex amplitude hologram is displayed. The hologram is determined by propagating a target image to the SLM plane with the central wavelength. Each reconstructed wave propagates to the eye-lens where the lens profile is multiplied to the incident complex amplitude. The transmitted wavefront arrives at the observation plane after propagation. Note that all propagation is applied by angular spectrum method with zero-padding. This holographic reconstruction is conducted for all sampled angles and wavelengths. Finally, we derive the final image in partially coherent holographic displays by synthesizing all observation images. In the synthesis, we consider the apodization of the aperture and spectral power distribution.

To extract speckle contrast, we reconstruct a white board image at the desired depth and calculate the synthesized image at the sensor plane. Before calculating the speckle contrast, the synthesized image is cropped at the center to mitigate the undesired effect such as vignetting. The average and the standard deviation of cropped image's intensity is estimated and the speckle contrast is determined. For resolution evaluation, we reconstruct a resolution chart that has a spoke radial pattern. The reconstructed images are also synthesized at the sensor plane. The synthesized image is Fourier transformed at this time to derive the contrast curve according to the spatial frequency. To calculate the transfer function, we also use the Fourier transform of the original target image. The normalized contrast curve of the synthesized image is divided by that of the target image. The result is considered as the transfer function of the partially coherent holographic display.

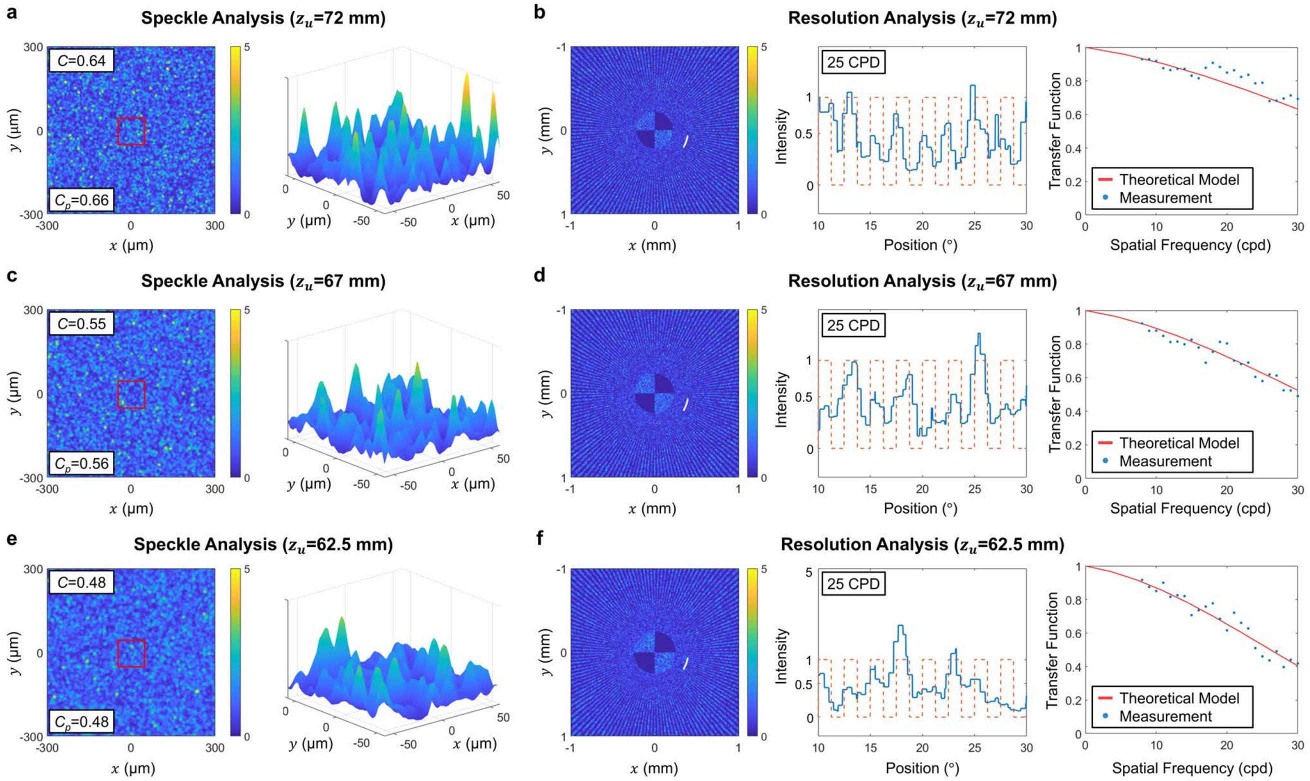

Supplementary Figure S3. Simulation results for the reconstruction depths of (a-b) 72 mm, (c-d) 67 mm, and (e-f) 62.5 mm. (a, c, e) Illustration of the speckle patterns and their magnified meshed charts for more intuitive visualization. (b, d, f) Resolution test of holographic reconstruction according to the light sources. Magnified intensity information along the white lines in the captured images and the transfer function calculated by Fourier transform of the intensity profile. Theoretical prediction of the speckle contrast and the transfer function are illustrated by C_p and red solid lines.

Additional Results

More simulation results to confirm the validity of the theoretical model is demonstrated in Fig. S3. In the figure, we present three more results for the reconstruction depths at 62.5 mm, 67 mm, and 72 mm from the eye-lens. As shown in the result, speckle contrast and resolution prediction fit well with the simulation. However, there is a little discrepancy between the simulation result and theoretical prediction. The primary reason for the difference is the approximation and assumption used in the theoretical model. Although we assume that the variation of the reconstruction depth or magnification is negligible, it contributes resolution degradation in some conditions. For instance, the discrepancy gets larger if the reconstruction depth is far from the SLM plane where the magnification effect gets stronger.

Supplementary Note 4: Implementation Details

Experiment Configuration

Figure S4 illustrates the schematic diagram of a benchtop prototype for partially coherent holographic displays, which integrates a laser diode (LD), a DMD, and an SLM. The LD (CPS532-C2,Thorlabs) with wavelength of 532 nm and power of 0.9 mW is collimated with a lens to function as a light source of holographic display. Unlike conventional holographic displays where the light source directly illuminates the SLM, the DMD is supplemented to increase the angle diversity of the LD as mentioned in the previous section. The DMD (DLP9500,Texas Instrument) supports the frame rate at most 23148 Hz, and the binary modulation of the 1920×1080 binary pixels with a size of 10.8 μm. Relay optics with a unit magnification are constructed with off-the-shelf lenses (150 mm focal length) to optically deliver the DMD to the SLM plane. In this relaying structure, a circular pinhole of 250 μm lies slightly away from the center of the frequency plane to eliminate a noise caused by the surface reflection from the DMD. The light source modulated by the DMD illuminates the liquid crystal on silicon (LCoS) reflective phase-only SLM (JD8714, Jasper display) with 3840×2160 pixels. The pixel pitch of the SLM is 3.74 μm so that the entire active area is 14.3 mm×8.1 mm. Finally, a holographic image is generated after passing through another set of relay optics

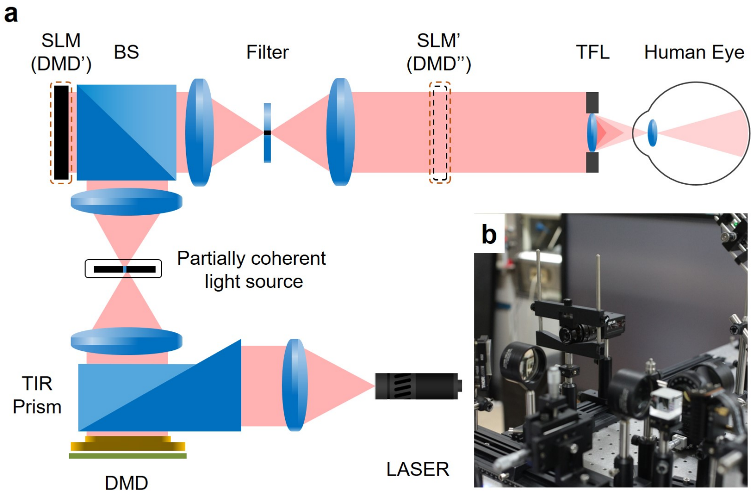

Supplementary Figure S4. (a) Schematic illustration of partially coherent holographic displays with extended depth of field (BS : beam splitter, TFL : tunable-focus lens). (b) A photograph of experimental setup is provided. The DMD projection system enables dynamic modulation of light source's angle diversity which can eventually imitate the partially coherent light source. In addition, it also enables local illumination for potential use in holographic display using tomographic synthesis. Note that there is additional tunable-focus lens for the volumetric reconstruction experiment, which increases the effective focal power of the eye-lens as similar to virtual reality systems.

with a spatial filter.

For the experimental setup used in Figs. 3 and 7, additional tunable-focus lens was used as illustrated in the figure. Note that the prototype demonstrated in Fig. 5 did not include the tunable-focus lens. Also, an LED source substituted the entire DMD projection system for the experiment of Fig. 7. The LED source aperture was located at the Fourier filter plane of the DMD system. In this setup, the holographic image was virtually floated by the tunable-focus lens (EL-10-30-TC-VIS-12D, Optotune), which works as an eye-piece lens with the optical power modulation range from 8.3 D to 20 D. The holographic image is captured with charge-coupled device (CCD) (GS3-U3-91S6C-C, FLIR) with a c-mount lens (F/6D, focal length of 25 mm).

Computer Generated Hologram

For the experiment demonstrated in Figs. 3 and 7, the complex hologram is calculated using the intensity and 8-bit depth profile in the dioptric unit. The 8-bit depth profile of the object is converted to the metric unit with the consideration of the display system. The number of sliced images were 32 where sampling follows SLM specifications(4K, 3.74 μm pitch). For experiment, we applied layer-based algorithm with single-viewpoint rendering geometry. We sliced 3D scene to multi-layered images using depth map, and superposed all sliced images' CGHs. Second, each CGH is calculated with step-wise depth propagation procedure. To be specific, angular spectrum method is adopted to propagate each depth difference and eventually acquire the desired hologram. The superposed CGH is converted to phase-only hologram by replacing amplitude as 1. Third, the generated hologram is processed according to the optical power of the tunable-focus lens. For the adaptive focus holographic display, the hologram is axially shifted with additional propagation distance so that the interested part of the volumetric scene is reconstructed at the central depth plane. Note that the appropriate optical power of the tunable-focus lens allows a user to experience accurate accommodation. Adaptive focus holographic near-eye display is a feasible and practical system as being implemented using off-the-shelf components. Besides, adaptive focus holographic displays have advantages compared to conventional gaze-contingent displays. While conventional gaze-contingent displays float a flat image at the desired depth, the proposed method reconstructs a volumetric image. By the volumetric reconstruction, appropriate accommodation is introduced to users if gaze difference is not significant. Thus, the gaze tracking is not necessarily much fast or accurate to be comparable with the human visual system for tolerant vergence-accommodation conflict mitigation. Gaze tracking latency and accuracy affect the resolution and speckle contrast of observed scenes rather than the accuracy of accommodation. It could be a significant advantage in a specific condition where the gaze tracking system's latency or refresh speed of displays is not enough to surpass the human vision limit.

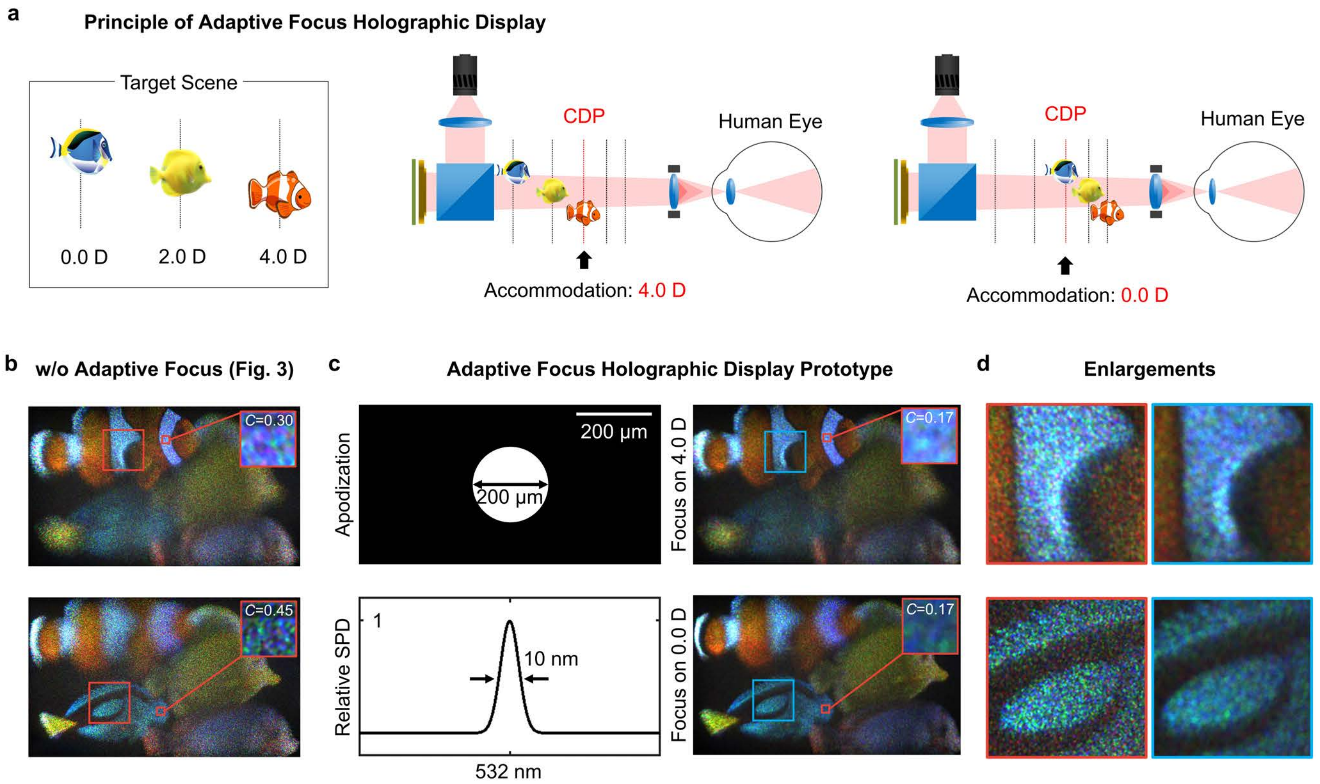

Supplementary Figure S5. (a) Schematic diagrams to illustrate the principle of adaptive focus coherent holographic displays. A tunable-focus lens floats the central depth plane (CDP) of hologram at the desired depth to provide accurate accommodation depth. (b) Experimental results of the partially coherent holographic display without adaptive focus method. (c) Illustration of light source specifications used for the adaptive focus holographic display. Photographs of the reconstructed holographic images are also presented according to the focal depths. (d) We compare the reconstruction results of two prototypes with or without the adaptive focus method. The proposed method enables more speckle reduction while maintaining high resolution. The speckle contrast values (C) are noted in the enlargements of display photograph. We present red/green/blue color images digitally combined from each color channel image. Tropical fish (royalty-free 3D model from Turbosquid) is used for the experiment.

Additional Results

We introduce a venturesome method to extend the depth of field while minimizing the speckle contrast. Figure S5(a) illustrates the schematic diagram how the adaptive focus holographic display reconstructs a volumetric scene. According to the gaze direction, the depth information of the scene is adjusted to reconstruct gazed objects mostly within the limited depth of field. The adjustment of the depth information is compensated by the tunable-focus lens to provide accurate accommodation. Figure S5(b-c) compare holographic displays with or without adaptive focus method. Figure S5 shows the light source specifications used for the adaptive focus holographic display. Note that the light source has a larger aperture so that the speckle contrast could be more reduced for a narrower depth of field. The experimental results in Fig. S5(d) show that the adopative focus holographic display has a reliable performance in wide depth of field. When the CDP is matched with the focal depth, the speckle contrast is less than 0.2 and the resolution reduction is also minimized. For example, the speckle contrast is reduced to 0.17 from 0.3 by adaptation of the TFL's focal power. The proposed system also has a merit compared to conventional gaze-contingent displays using a 2D display. Despite the narrow depth of field, the holographic reconstruction provides a tolerance for focus tracking error which has been a bottleneck of gaze-contingent displays.

We present additional experimental results in Fig. S6 to show that the light source optimization is a versatile method. The light source optimization can be combined with the temporal superposition of independent speckle patterns exploiting the fast frame rate of the SLM[2,3]. Figure S6 demonstrates the experimental results that digitally combine multiple reconstructed images. Each reconstructed image is sequentially captured by refreshing the CGH on SLM for independent speckle generation. According to the frame number (N_f) for the digital synthesis, the speckle contrast is reduced to $1/\sqrt{N_f}$. As demonstrated in the results, the speckle contrast is further reduced when the optimal source is applied. Also, the resolution of the synthesized

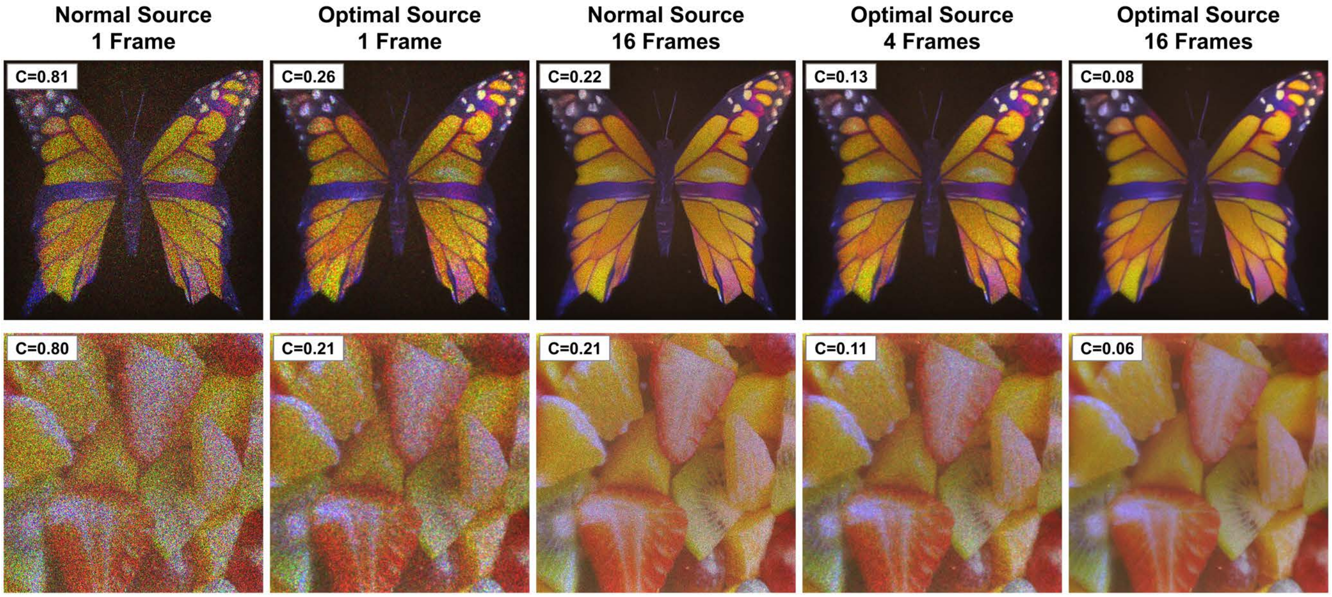

Supplementary Figure S6. Experimental results to illustrate the trade-off between the frame numbers for speckle superposition and the speckle contrast. The optimal source allows holographic displays to further reduce the speckle contrast regardless of the frame numbers. We present two different red/green/blue color images digitally combined from each color channel image. Butterfly (purchased 3D model from Free3D) and fruit (purchased imagery from shutterstock) are used for the experiment.

images is well preserved. Compared to the normal source, the optimal source allows holographic displays to achieve much more speckle reduction while maintaining resolution and frame rates. In summary, the optimal source alleviates the trade-off among the speckle contrast, resolution, and frame rate. In this experiment, the holograms were generated by the primitive method. We used two different images (butterfly: source imagery from publisher "Free3D", fruit: source imagery from publisher "shutterstock") for the experiment.

References

1. Goodman, J. W. *Introduction to Fourier Optics* (Roberts and Company Publishers, USA, 2017).

2. Takaki, Y. & Yokouchi, M. Speckle-free and grayscale hologram reconstruction using time-multiplexing technique. *Opt. Express* **19**, 7567–7579 (2011).

3. Mori, Y., Fukuoka, T. & Nomura, T. Speckle reduction in holographic projection by random pixel separation with time multiplexing. *Appl. Opt.* **53**, 8182–8188 (2014).
